# Key sources and seasonal dynamics of greenhouse gas fluxes from yak grazing systems on the Qinghai-Tibetan Plateau

**DOI:** 10.1038/srep40857

**Published:** 2017-01-20

**Authors:** Yang Liu, Caiyu Yan, Cory Matthew, Brennon Wood, Fujiang Hou

**Affiliations:** 1 State Key Laboratory of Grassland Agro-ecosystems, College of Pastoral Agriculture Science and Technology, Lanzhou University, Lanzhou, 730020, China; 2 Institute of Agriculture and Environment, Massey University, Private Bag 11-222, Palmerston North, New Zealand

## Abstract

Greenhouse gas (GHG) emissions from livestock grazing systems are contributing to global warming. To examine the influence of yak grazing systems on GHG fluxes and relationships between GHG fluxes and environmental factors, we measured carbon dioxide (CO_2_), methane (CH_4_) and nitrous oxide (N_2_O) fluxes over three key seasons in 2012 and 2013 from a range of potential sources, including: alpine meadows, dung patches, manure heaps and yak night pens, on the Qinghai-Tibetan Plateau. We also estimated the total annual global warming potential (GWP, CO_2_-equivalents) from family farm grazing yaks using our measured results and other published data. In this study, GHG fluxes per unit area from night pens and composting manure heaps were higher than from dung patches and alpine meadows. Increased moisture content and surface temperature of soil and manure were major factors increasing CO_2_ and CH_4_ fluxes. High contributions of CH_4_ and N_2_O (21.1% and 44.8%, respectively) to the annual total GWP budget (334.2 tonnes) strongly suggest these GHG other than CO_2_ should not be ignored when estimating GWP from the family farm grazing yaks on the Qinghai-Tibetan Plateau for the purposes of determining national and regional land use policies or compiling global GHG inventories.

Increased atmospheric concentrations of trace greenhouse gas (GHG), in particular carbon dioxide (CO_2_), methane (CH_4_) and nitrous oxide (N_2_O), have been identified as the main contributors to global warming^1^. Discovering the sources and sinks of these gases in terrestrial ecosystems has therefore become an important aim for global research. Considerable scientific effort has focused on grasslands. Comprising approx. 40% of the globe’s terrestrial ecosystem^2^,^3^, grassland plays a major role in the biosphere-atmosphere exchanges of the major GHG.

Pastoral farming is an important human activity that significantly affects grassland ecosystems. Livestock grazing alters the exchange of carbon and nitrogen between the biosphere and the atmosphere. Key processes include animal feed intake, excreta deposition (dung and urine) and trampling of the ground. The proportion of ingested nutrients that are retained in the body tissue of grazing livestock is small. Most of the mineral nutrients consumed are excreted in faeces and urine^4^. Ruminants may excrete 75–95% of the nitrogen (N) consumed^5^, though increased stocking rate reduces soil C sequestration potential and C and N dynamics are intertwined^6^. High N loading generated by an increased stocking rate and the associated urine and dung patches, to a much lesser extent, is of particular environmental concern because of the potential for leaching of nitrate N below the root zone of pasture plants, with subsequent degradation of ground water. With increased soil N loading, soil nitrate N is subject to transformations that can yield nitrogen oxides, including N_2_O under chemically reducing conditions, such as when soil oxygen is depleted by high moisture levels. A single urine deposit by European cattle (*Bos taurus*) may yield the equivalent of 400–500 kg N ha^−1^ and over 800 kg elemental K ha^−1^ applied to the soil^7^. The pool of available nitrogen and carbon added to the soil by livestock excreta provides substrate for the production of N_2_O as well as CO_2_ and CH_4_ by soil microorganisms^8^. Deposition sites of animal excreta, particularly urine, during grazing have been referred to as “hot spots” for gaseous N losses and N leaching to air and water due to the high nutrient loading in these areas^9^.

As the largest and highest grassland unit on the Eurasion continent, the Qinghai-Tibetan Plateau (QTP) covers an area of approx. 2.5 million km^2^ and it is often referred to as the ‘Third Pole’ (mostly situated at 4000 m above sea level), playing a critical climatic role, governing the Asian monsoon and it is the source of river systems that provide water to some 20% of the world’s population^10^,^11^,^12^,^13^. Alpine meadow, which is the major pastureland on the plateau, covers 35% of the plateau area. There are in excess of 13.3 milion domestic yaks and 20 thousand wild yaks living on QTP^14^, large amounts of yaks excreta (dung and urine) are deposited onto the alpine grassland which can make a huge GHG emsssions. Furthermore, unlike the paddock-based systems of Australian and New Zealand farmers, herdsmen on the QTP typically practise open grazing in alpine meadows during the day and then bring their yaks back to outdoor pens close to their dwellings during the night^15^. Prolonged excreta accumulation and animal trampling in these pens creates conditions for alternating wet and dry hypoxic fermentation, which may be a significant source of GHG emissions. Research has found that US feedlots are significant N_2_O sources^16^, suggesting that the impact of yak night pens requires investigation. About 40% of the total yak excreta is deposited in the night pens^17^. Yak dung in the pens is regularly collected and stored in manure heaps for drying for use as household fuel, and such heaps may also be sources of GHG. Although some studies have reported that alpine meadows on the QTP acts as a weak CH_4_ sink during the growing season^18^, excreta patches deposited onto grassland as “hotspot” of CH_4_ and N_2_O emissions^14^, as well as feedlots as the significant N_2_O sources^16^; so far, they have not taken into account of the effects of excreta patches, expecially manure heaps and night pens, on GHG emissions in the whole farm practices.

Thus, the overall objective of our study was to quantify GHG emissions from yak farming, taking account in the measurement protocol of farm practices such as use of night pens and manure heaps, as well as evironmental factors. In this way the study was expected to expand the limited published data on GHG emissions from family farm grazing yaks on QTP in China and contribute additional data for use in global GHG inventories. The specific aims of this study were to (1) observe temporal variation in CH_4_, CO_2_ and N_2_O fluxes of key sources (alpine meadows, dung patches, manure heaps and night pens) under yak grazing systems across different seasons(early growing, peak growing and non-growing season); (2) evaluate relationships between GHG fluxes and physico-chemical factors (i.e. surface temperature (ST), moisture content (MC), total organic carbon (TOC), total nitrogrn (TN), microbial bimass carbon (MBC) and microbial biomass nitrogrn (MBN)); (3) estimate the total annual GWP (CO_2_-equivalents) and the contribution of each gas from a typical family farm grazing yaks.

## Results

### GHG fluxes

**Figure 1:**
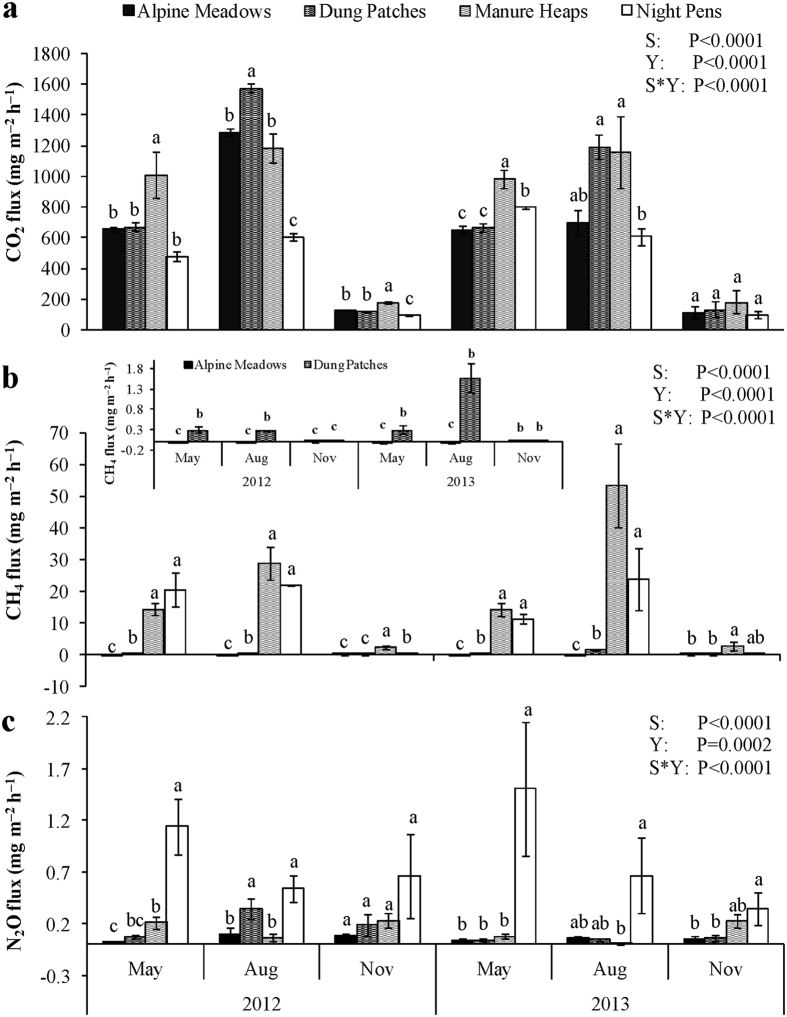
Seasonal changes in CO_2_, CH_4_ and N_2_O fluxes from the different GHG sources. S, Source; Y, Season (Year/Month); S*Y, the interaction between source and season (Year/Month). Data are presented as the mean ± 1 SE (n = 9). Columns with the same letters have no significant seasonal differences (P > 0.05). The p values presented in the figures are the significance levels for source, season and their interaction in the GLM model. Negative values for CH_4_ fluxes represent uptake by soil/manure.

Fluxes of CO_2_ varied significantly by source and season, with differences between sources in their seasonal pattern (Fig. 1a), as indicated by statistically significant interaction between source and season in ANOVA. Each source showed rising CO_2_ fluxes from the early growing season (May) to a high point in the peak growing season (August), then lower but continuing flux in the non-growing season (November). CO_2_ emitted from the manure heaps was significantly higher than from the other sources in May and November 2012 and May 2013 (Minimum Significant Difference(MSD) = 298.9, 18.6 and 132.2 respectively, *P* < 0.001). However, CO_2_ emissions were significantly higher from dung patches than other sources in August 2012 (MSD = 200.5, *P* < 0.0001) and from both dung patches and manure heaps in August 2013 (MSD = 505.9, *P* = 0.0044), with no significant difference detected among sources in November 2013 (MSD = 194.3, *P* > 0.05).

Seasonal patterns of CH_4_ fluxes differed strongly among measured sources, as indicated by highly significant interaction effects between source and season (*P* < 0.0001). In general alpine meadow soils were slightly methanotrophic, while dung patches had small methane effluxes. Manure heaps and night pens generated substantial CH_4_ effluxes, especially in the higher temperatures of the peak growing season, but with some variability between years (Fig. 1b). Fluxes in the alpine meadows were negative in May and August 2012 and 2013 (i.e. meadows were methanotrophic), with positive values measured for the other three sources at this time. Positive values were also observed for each source in November (the non-growing season) during the two year experimental period. With the exception of the alpine meadows, CH_4_ fluxes rose from May to August and then dropped in November. Fluxes from manure heaps and night pens were significantly higher than from the alpine meadows and dung patches in May and August 2012 and 2013 (MSD = 0.51, 0.33, 0.62 and 1.26 respectively, *P* < 0.0001). However, CH_4_ fluxes were significantly higher from the manure heaps in November 2012 and 2013 than from the other sources (MSD = 0.58 and 1.17, *P* < 0.0001 and *P* = 0.0107). Emissions from dung patches were significantly higher than the alpine meadows in May and August 2012 and 2013, with no significant difference detected with the alpine meadows in November 2012 and 2013.

For N_2_O fluxes the notable feature of the data was that emissions from night pens were always numerically highest among the sources, though not always statistically so (Fig. 1c). N_2_O fluxes also showed temporal variability as indicated by statistical interactions between GHG source and season. Fluxes from dung patches were statistically higher than from the alpine meadows during the measurement made in August 2012 (MSD = 1.41, *P* < 0.0001), but apart from this, no consistent pattern was observed.

### Physico-chemical properties

**Figure 2:**
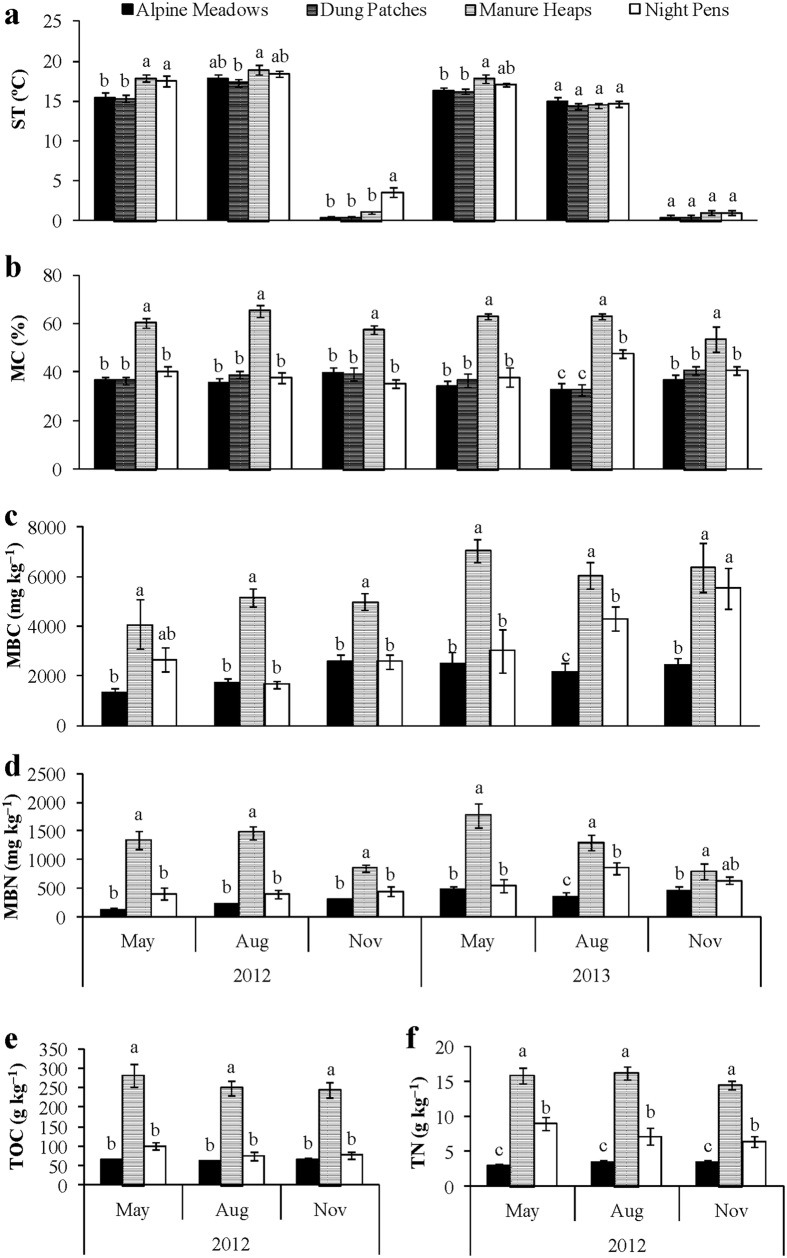
Seasonal variation in the physico-chemical properties of soil and manure for the GHG sources. Data are presented as means ± 1 SE (n = 9). Columns with same letters have no significant differences (P > 0.05) for each season (Year/Month). ST, surface temperature; MC, moisture content; TOC, total organic carbon; TN, total nitrogen; MBC, microbial biomass carbon and MBN, microbial biomass nitrogen. Alpine meadows and Dung patches share the same values of MBC, MBN, TOC and TN; TOC and TN were measured for 2012 only.

ST showed an expected seasonal pattern during the two year experimental period, with only minor differences between sources; manure heaps and night pens being on some occasions a little warmer than the surrounding meadows (Fig. 2a). MC of alpine meadow soils was typically around 40% (range 32.6–47.5%) with no indication of seasonal variation and no elevation at dung patch sites or in night pens. Manure heaps had higher MC values (53.7–65.4%) (Fig. 2b). As would be expected, manure heaps had higher MBC and MBN contents than other sampled GHG sources, although there was MBC and MBN accumulation in night pens in 2013. Neither MBC nor MBN exhibited significant seasonal variation (Fig. 2c,d). TOC values measured in 2012 only were 258.5, 84.1 and 64.3 g kg^−1^ for manure heaps, night pens, and alpine meadow soil, respectively (Fig. 2e); while corresponding TN values were 15.5, 7.5 and 3.3 g kg^−1^, respectively (Fig. 2f). Neither TOC nor TN varied significantly by season.

### Key factors associated with GHG fluxes

Pearson correlation analysis was used to overview the relationships between GHG fluxes and the physico-chemical indicators measured (Table 1). Fluxes of the three GHG were only weakly correlated with each other. CO_2_ and CH_4_ fluxes (but not N_2_O) are increased with increased ST. CH_4_ fluxes were correlated with all the parameters and the strongest correlation was with TN. N_2_O fluxes were weakly but significantly negatively correlated with MC, MBC and MBN. Quantification of the relationships by multiple linear regression (Table 2) largely reflected these patterns but did also highlight a role of TN in N_2_O flux, likely reflecting high N_2_O efflux from night pens (Fig. 1), and much higher TN values for night pens than for alpine meadows.

### Data imported from other studies

The GHG fluxes from yak urine patches which were not directly measured in our study (since data were available from other studies at other QTP sites with similar vegetation), need to be estimated to complete the inventory of the total annual GWP from a family farm grazing yaks, The results of averaged GHG fluxes from yak urine patches, obtained with the model of Lin *et al*.^14^ are presented in Table 3.

### Integration of GHG Sources to estimate GWP

A relational diagram of GHG sources for QTP yak grazing systems is presented in Fig. S1. Our methodology for estimating the total annual GWP from a family farm grazing yaks, followed the organisation of the relational diagram and used our measured results (i.e. GHG fluxes from alpine meadows, dung patches, night pens and manure heaps) and other published data in some cases, as described in the “Methods” section, below. The results of our family-farm-level integration of GHG fluxes (two year averaged data) are presented in Table 4. It is seen that total annual GWP (CO_2_-eq.) from a typical QTP family farm (average farm size 83 ha, yak population 87 head; stocking rate approximate 1.05 yaks ha^−1^) is estimated at 334.2 tonnes. Of the annual total GHG emissions, CO_2_ release accounted for approximately 34.1% of emissions, while CH_4_ and N_2_O emissions contributed approximately 21.1% and 44.8%, respectively. By season, the GWP during the non-growing season (winter) was 600.7 tonnes CO_2_-eq. (Table 4), however, GWP of the non-growing season was offset by ecosystem CH_4_ and CO_2_ uptake during the early growing and peak growing season of −119.6 and −147 tonnes CO_2_-eq, respectively.

## Discussion

CO_2_ fluxes seasonally measured in this study were respiration from alpine meadows, fluxes from dung patches in the meadows and manure heaps amassed by farmers, and fluxes from night pens. Respiration from the alpine meadows is made up of soil heterotrophic respiration and the autotrophic respiration of vegetation from both above and below the ground (plant height in the meadows was approximately 50–70 mm). The fluxes measured from the yaks’ night pens are primarily due to soil heterotrophic respiration and yak excreta (with no significant plant respiration in the pens due to extensive trampling of the ground). The results show a clear pattern of seasonal variation in CO_2_ fluxes from all four GHG sources in the yak grazing systems of the studied farms on the QTP. Highest emissions were in the peak-growing season for plants (August) when temperatures were relatively high, lowest in the non-growing season (November) when temperatures were relatively low. This is an expected result, given that previous studies have shown that soil temperature and moisture are dominant environmental variables controlling seasonal changes in CO_2_ emission^19^. Our study found that surface temperature and soil water content are also key factors affecting these emissions (Tables 1 and 2). Urea in animal urine undergoes hydrolysis catalysed by the enzyme urease to form (NH_4_)_2_CO_3_, with the resulting carbonate hydrolysis producing CO_2_^20^; previous studies have thus found pulses in CO_2_ emission following urine application^21^,^22^. Given these findings we might expect significant emissions from the night pens with their abundant deposition of yak urine. In our study, however, the CO_2_ effluxes from night pens were lower than from the other sources. It seems likely that respiration from pen soil was inhibited due to anaerobic conditions and the lack of vegetation following yak trampling. In addition, we found that fluxes from dung patches and manure heaps tended to be either equal to, or exceed those from alpine meadows and night pens across all three seasons (Fig. 1). These higher levels of CO_2_ emission may be due to higher soil organic carbon and microbial community activities, both of which may contribute to increased respiration.

The CH_4_ fluxes presented the net flux between consumption and production by methanotrophic and methanogenic microbes. A previous study^18^ has reported that alpine meadows on the QTP are a weak CH_4_ sink, with average uptake fluxes −59.2 ± 3.7 μg CH_4 _m^−2 ^h^−1^ during the growing season. Our research observed that alpine meadows absorbed CH_4_ in the early and peak growing seasons, but emitted it in the non-growing seasons for both years sampled. The mean CH_4_ fluxes in May, August and November were −20.7 ± 5.3, −27.3 ± 3.0, 3.5 ± 7.4 μg CH_4 _m^−2 ^h^−1^, respectively, during the two year period of the experiment. Given the extensive duration of the winter period (197 days), this is a biologically significant result that should be incorporated in GHG inventories of the plateau. Yak dung deposition significantly increased CH_4_ emissions, a finding attributable to dissolved CH_4_, large microbial populations, highly degradable organic compounds and the anaerobic condition of the fresh patches^23^,^24^. The MC of soil strongly controls CH_4_ dynamics given the increases in production due to anaerobic conditions^25^. Our study shows the significantly higher MC of the manure heaps (approximately 60%), thus higher CH_4_ fluxes were observed from manure heaps. Although the night pens contain high dung levels, across all seasons they tended to emit less CH_4_ per unit area than the manure heaps. Increased soil pH after livestock urine deposition significantly reduces CH_4_ oxidation rates^19^,^26^. Moreover, soil NH_4_^+^ -N content may limit the capacity of soils to take up CH_4_ as it can inhibit the activity of methanotrophs^27^,^28^. Previous research has found that with increasing soil applications of NH_4_^+^ -N from 13 to 96 kg N ha^−1^ the amount of CH_4_ oxidation fell by amounts ranging from 31 to 97%^26^. Yak urine excreted to the night pens contains both water and urine-N and the combined effect of these two factors may both promote CH_4_ production and inhibit its consumption by soil in the night pens. Our study thus suggests that the relationship between CH_4_ emission and soil NH_4_^+^ -N requires further research.

N_2_O fluxes result from biological processes of nitrification and denitrification. In general, nitrification involves the aerobic microbial oxidation of ammonia into nitrate (NO_3_^−^), denitrification of the anaerobic microbial reduction of NO_3_^−^ to NO, N_2_O, and N_2_. These processes result in N_2_O emissions as an intermediate by-product. Our research shows that night pens were consistently the highest N_2_O emitters and that all sources exhibited significant seasonal variations. These results are attributable to the spatially and temporally highly variable character of nitrification processes. Nitrification and denitrification depend on soil and manure moisture and pH levels, temperature, organic matter, NO_3_^−^ and NH4^+^ content and the nature of the microbial community, as well as a range of other factors^16^. The combined effect of these factors clearly favours N_2_O emissions from pens more than from other sources measured, and from pens in the early growing season in particular, though their impact on seasonal fluxes from the other sources is complex. The results show that N_2_O fluxes were higher from dung patches than alpine meadows throughout 2012. Moreover, fluxes were higher from manure heaps than alpine meadow only in the 2012 early growing season and the 2013 non-growing season. These findings are difficult to interpret, although they are generally consistent with prior studies reporting that animal dung application stimulates N_2_O emission in the field^29^,^30^,^31^,^32^. Dung patches and heaps are likely to have high denitrification rates due to internal anaerobic conditions, combined with high levels of moisture, TOC, TN, MBC and MBN. Nitrogen losses from NH_3_ volatilization and N leaching can significantly reduce the denitrification potential of sources^32^. The relatively lower N_2_O emissions from manure heaps during the peak growing season may thus be due to the warm and dry measurement conditions. These conditions can be optimal for NH_3_ volatilisation losses, leaving less inorganic N in the manure for N_2_O formation and emission^33^,^34^, although this assumption requires testing by further research. The negative correlation between N_2_O fluxes and MBC or MBN is counterintuitive and we were not able to unravel the reason, but it is likely to relate to the influence of unusual data like those for night pens on the correlation patterns. Our research showed that N_2_O fluxes were higher from the yak night pens than from any of the other sources in all seasons. This result suggests that night pens provide good conditions for soil nitrification and denitrification due to the combined effect of microbial nutrition substrates derived from urine deposition and the anaerobic conditions produced by yak trampling. Compaction due to animal trampling promotes anaerobic conditions by reducing soil porosity, hydraulic conductivity and root penetration^35^,^36^. Given these observed effects of concentrating yaks in night pens, and the fact that the area of night pens on the QTP will expand with increasing yak populations, their N_2_O fluxes should not be ignored when estimating GHG emissions from yak grazing systems on the QTP.

Practically speaking, understanding the role of the QTP in global warming requires a thorough analysis of GHG fluxes generated by its yak grazing systems. The plateau is not simply a natural landscape; it is populated by farmers whose techniques are both ancient and rapidly changing. These nomadic pastoralists and their livestock are at the forefront of ecological degradation on the plateau and are the target of policies aimed at reversing this degradation. We have investigated GHG fluxes from four key sources in yak grazing systems on nine farms, and to address logistical issues from the number of samples to be collected, we have adopted some measurements of other researchers at similar sites (notably GHG fluxes from urine deposition in meadows and rumen emission of CH_4_) rather than repeat those measurements in our own study. Our nine farms were initially chosen to represent a range of stocking rates but after data were collected it became apparent that spatial heterogeneity within farms had resulted in data variability such that it was more meaningful to average results for all nine farms. In hindsight this has provided a strength to the study because averaging of nine replicates is a sound way to deal with variable data. Even so, for a number of reasons we believe confirmation through additional data collection would be desirable. Moreover, we lack full seasonal data for all significant GHG sources and must rely on interpolative estimates. Our synthesis of GWP for the yak grazing systems thus requires cautious interpretation, but should provide a useful first estimate of the comparative size of these fluxes, pending further research on this topic.

These data indicate (Table 4), that in the QTP yak grazing family farm systems N_2_O is more important on an annual basis than CO_2_, and that the carbon sink activity of the alpine meadows during the early and peak growing seasons has a GWP mitigation effect, but there is a substantial carbon respiration during the non-growing season with additional GWP from CH_4_ and N_2_O emissions, which while highest in the non-growing season, also continues through the early and peak growing seasons. To place these observations in context, Merbold *et al*.^37^ reported CO_2_ emission from snow covered alpine meadows in Switzerland to be 0.77 μMol m^−2^ s^−1^, which converts to just 3.54 tonnes for their 121 day study period from 1 December to 31 March. The same authors reported a small CH_4_ uptake and N_2_O emission of 0.23 n Mol m^−2 ^s^−1^, which equates to just 0.315 tonnes CO_2_-eq. in their 121 day winter measurement period. It seems reasonable to assume that the transfer of N via urine to night pens accounts for the high GWP contribution of N_2_O in the QTP data, compared to the Swiss data. A hypothesis for further study is that the present defoliation intensity and warming on the QTP have combined to create a net annual loss of soil carbon, as indicated by data of Yuan and Hou^38^. The difference between the results of these two studies in Switzerland and on the QTP highlights the importance of obtaining site specific data for GWP inventory calculations, and shows that management of the QTP, with its very large land area, will be a significant element in determining regional land use policies and will have measureable global impact.

## Conclusions

The single largest source of GWP identified in this study was CO_2_ emission by alpine meadow soils in winter, and a hypothesis for further study is that the defoliation intensity from an average stocking rate of 1.05 yaks ha^−1^ on family farms on the QTP, coupled with a climatic warming trend, is resulting in a release of sequestered soil C. The GHG fluxes of N_2_O and CH_4_ from night pens and composting manure heaps per unit area were expectedly high, with yak night pens being an important N_2_O emission source, and when calculated as whole-farm quantities exceeded the annual GWP from CO_2_ emission by a factor of approximately 2. Total annual GWP (CO_2_-eq.) from the typical 83 ha family farm with an average stocking rate of 1.05 yaks ha^−1^ is 334.2 tonnes. The yak grazing family farm system has a GWP mitigation status during the early and peaking growing seasons, but is a large carbon source during the non-growing season. The contributions of CH_4_ and N_2_O (21.1% and 44.8%, respectively) to the annual total GHG budget strongly suggest these GHG should not be ignored when estimating GWP (CO_2_-eq.) in the grazing alpine meadows on the QTP for the purposes of determining national and regional land use policies or compiling global GHG inventories.

## Methods

### Study site

The study investigated yak grazing farms located 5–10 kilometres from Azi Research Station, Maqu County, Gansu Province, China, which is located in the eastern part of the QTP at longitude 101°53′E, latitude 33°58′N. Average elevation is 3700 m above sea level, with a cold, humid climate typical of the Tibetan Plateau^39^. Annual average temperature is approximately 1.2 °C, with lowest monthly average temperatures around −10 °C in December to February and the highest monthly average temperatures just below 12 °C in June to August each year. Annual average precipitation is 620 mm, mainly concentrated within the growing season from May to September. Vegetation is dominated by typical alpine meadow types: clonal *Kobresia* species and *Carex kansuensis* (Cyperaceae), *Festuca ovina, Poa poophagorum, Elymus nutans* and *Agrostis* species (Poaceae), *Saussurea* species (Asteraceae) and *Anemone rivularis* (Ranunculaceae)^40^,^41^.

### Experimental design

Nine typical yak grazing farms were selected to measure GHG fluxes and to explore the influence of soil and manure physico-chemical properties. In our study, each of the 9 yak farms was taken as an independent experimental unit and considered as a grazing system defined by its family ownership and related statistics (Table S1) and with a hierarchically related set of major GHG sources (Fig. S1).

For analytical purposes, the yak grazing systems are conceptualised as a coupling of soil, plant and animal related processes. Plant related processes are primarily associated with the pasture plant community but also involve the fertilisation of alpine meadows by animal excreta. Animal related processes include emissions by the yaks themselves, GHG sources arising from human management of these animals, and the direct losses from excreta deposited on pasture. GHG emissions from urine patches in alpine meadows and from rumen fermentation of yaks were not directly measured in this study, though relevant findings from previously published research for these sources will be included in the paper’s discussion of results. We follow the accepted methodology for dealing with respiratory emissions of CO_2_ by animals in GHG accounting, namely these emissions are zero-rated. This is because herbage eaten by animals represents CO_2_ recently acquired by photosynthesis and whether respired by animals or by eventual decomposition of uneaten herbage is returned to the atmosphere without involvement in any longer-term C-sink^42^. Direct N_2_O emissions from yaks were also excluded as these were considered to be negligible^43^.

### Measurement of GHG emissions

For the purposes of GHG measurement, three seasons have been identified on the plateau: early growing season, peak growing season and non-growing season^44^. In order to capture data for these three periods, we sampled CO_2_, CH_4_, and N_2_O fluxes of the various sources everyday (except for rainy weather) in May, August and November in 2012 and 2013. In order to determine season duration for estimation of annual GHG emission totals from measured seasonal values, we examined the 2013 daily temperatures and precipitation recorded at the Azi Research Station (Fig. S2). From those data we judge that:

1. The early growing season runs for 61 days from mid-April to mid-June (average temperature 6.9 °C and total precipitation 107.5 mm);
2. The peak growing season runs for 107 days from mid-June to the end of September (average temperature11.1 °C and total precipitation 368.1 mm); and
3. The non-growing season runs for 197 days from October to mid-April (average temperature −2.7 °C and total precipitation 56.9 mm).

GHG fluxes were measured using the static opaque chamber methods following the guidelines from previous research^25^,^45^,^46^,^47^,^48^,^49^,^50^. These chambers were constructed as described by Lin *et al*.^14^. Each chamber in a seat with a water groove to make the chamber seal with the seat airtight was inserted into the soil or manure to a depth of 50 mm. A battery-operated fan was installed on the top wall of each chamber to mix the air when the chamber was closed. Gas samples were drawn through a three-way stopcock, using a 60 ml syringe, and then transferred for storage into 500 ml aluminium foil gas-collecting bags (China Dalian Delin Gas Packing Co., Ltd). Four gas samples of approximately 250 ml were taken in each chamber at four time intervals for each sampling event (0, 10, 20 and 30 min) from 9:00 am at local time to represent daily mean flux^51^. Temperatures inside the chamber and at soil or manure depths of 50 mm were also recorded on each sampling occasion. A CH_4_/CO_2_ analyser with syringe injection (DLT-100, Model No. 908-0011-0001) was used for simultaneous CH_4_ and CO_2_ analysis, and a N_2_O/CO analyser (Model No. 908-0015-0000) was used for simultaneous N_2_O analysis. The fluxes were calculated according to the equation described by Song *et al*.^46^, with modifications made for QTP conditions as follows:


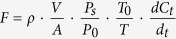


where *F* is the gas flux (mg m^−2 ^h^−1^), ρ is the gas density under standard conditions (CO_2_, CH_4_, N_2_O; 1.977, 0.717, 1.978 kg m^−3^, respectively), *V* is chamber volume (m^3^), *A* is the base area of the chamber (m^2^), *P*_*s*_ is the atmospheric pressure (kPa) of the sampling sites, *P*_*0*_is atmospheric pressure under standard conditions (101.325 kPa), *T*_*0*_is the temperature under standard conditions (273.15 K), *T* is the temperature inside the chamber (K), and *dC*_*t*_/*d*_*t*_ is the average rate of concentration change with time.

### Analysis of soil and manure physico-chemical properties

Soil and manure samples were collected at each measurement event. For each of the sources (soil or manure), surface layer temperatures (ST) were measured at 50 mm depth and the other physico-chemical properties from sample material from 0–100 mm depth. The moist fresh samples were analysed to determine their moisture content (MC), microbial biomass carbon (MBC) and microbial biomass nitrogen (MBN). The samples were then air-dried to measure total organic carbon (TOC) and total nitrogen (TN). MC was analysed by oven-drying the samples at 105 °C to constant weight. TOC was determined by the wet combustion method, with K_2_Cr_2_O_7_ and concentrated H_2_SO_4_ at 170–180 °C, and TN in the digest was determined using a continuous flow analyser (FIAstar 5000 Analyzer). MBC and MBN were determined using the chloroform fumigation-extraction method^52^,^53^. The samples were conditioned for 7 days at 25 °C while maintaining 40% of their total water holding capacity. Then the fumigated and non-fumigated samples were extracted with K_2_SO_4_ solutions (0.5 mol L^−1^) with a 1:4 (weight to volume) soil/manure-to-extractant ratio. MBC and MBN concentrations were calculated as the differences in total extractable C and N between the fumigated and non-fumigated samples, with the conversion factors set at 2.64 for biomass C and 1.85 for biomass N^53^.

### Data adopted from other studies

To complete the inventory of the annual GHG fluxes from the yaks grazing family farm level (Fig. S1), the GHG fluxes from rumen activity of yaks and from their urine patches which were not directly measured in our study, needed to be estimated. Ruminant rumen fermentation is an important source of CH_4_. In our study, we use the emission factor 81.4 g CH_4_ day^−1^ to estimate the CH_4_ emission from yaks, based on assumptions of 175 kg live-weight and an intake of 3.78 kg DM/day^54^.

An independent variable linear model from Lin *et al*.^14^ was chosen to estimate averaged GHG fluxes from yak urine patches. Soil surface temperature (0–50 mm) and WFPS (0–100 mm) data invested at sampling day were used in this model to calculate the averaged CH_4_, CO_2_ and N_2_O fluxes for each season.

Also, the CO_2_ fluxes measured using the static opaque chamber method described above represent the respiration of alpine meadows ecosystems. To estimate GWP of alpine meadows, the CO_2_ consumption by plant photosynthesis also needs to be accounted for Chen *et al*.^55^ reported net carbon ecosystem exchange (NEE) during the growing season (total 175 days) of −192.11 g C m^−2^ (that is to say −167.72 mg CO_2 _m^−2 ^h^−1^) for free-range grazing in a meadow grassland on Tibetan Plateau. We adopted this value of −167.72 mg CO_2 _m^−2 ^h^−1^ to estimate GWP during early growing season and peak growing season in our study, while still using our measured respiration values in non-growing season when plant photosynthesis can be assumed to be zero.

### Integration of GHG data to estimate GWP

Following the methodology of other published studies^56^,^57^, the combined impact of different sources and GHG is expressed as a GWP in CO_2_ equivalents (CO_2_-eq.) based on GWP factors compared to CO_2_ of 25 times for CH_4_ and 298 times for N_2_O for a time horizon of 100 years^1^. Total CH_4_ fluxes from yaks are the product of emission factor, averaged yak population and season days. However, total GHG (CO_2_, CH_4_ and N_2_O) fluxes for these key sources (Alpine meadows, Dung patches, Urine patches, Manure heaps and Night pens) at average family farm level for each season were simplified calculated using the equation as follows: Total GHG Fluxes = mean GHG fluxes × area × season days.

### Statistical analysis

All statistical analyses were conducted using SAS 9.3 (SAS Institute Inc., Cary, NC, USA), with significance levels set at *P* < 0.05. A goodness-of-fit test (Shapiro-Wilk test) was used to test data distributions and confirm normality. Data for soil and manure physico-chemical properties and for GHG fluxes were analyzed using ANOVA (Proc GLM), followed by Tukey’s HSD tests. Prior to ANOVA, the data on CH_4_ and N_2_O fluxes were log-transformed (ln(*x*)), but the means calculated from the original data are presented. A general linear model (Proc GLM) was applied to determine the effects of source and season and their interactions (source x season) on GHG emissions. To test the correlations between GHG fluxes and physico-chemical properties, Pearson correlations (Proc CORR) was performed. Multiple linear regression analysis (Proc REG) of GHG fluxes on the physico-chemical indicators was performed.

## Additional Information

**How to cite this article**: Liu, Y. *et al*. Key sources and seasonal dynamics of greenhouse gas fluxes from yak grazing systems on the Qinghai-Tibetan Plateau. *Sci. Rep.* **7**, 40857; doi: 10.1038/srep40857 (2017).

**Publisher's note:** Springer Nature remains neutral with regard to jurisdictional claims in published maps and institutional affiliations.

## Supplementary Material

Supplementary Information

## Figures and Tables

**Table 1:** Pearson correlation analysis of GHG fluxes and key physico-chemical indicators.

	CO_2_	CH_4_	N_2_O	ST	MC	TOC	TN	MBC	MBN
CO_2_	1								
CH_4_	0.282**	1							
N_2_O	−0.062	0.123	1						
ST	0.746**	0.447**	0.036	1					
MC	0.135*	0.571**	−0.160*	0.066	1				
TOC	0.157	0.583**	−0.163	0.083	0.852**	1			
TN	0.082	0.728**	0.030	0.134	0.854**	0.902**	1		
MBC	−0.084	0.396**	−0.168*	−0.10	0.686**	0.663**	0.721**	1	
MBN	0.151*	0.609**	−0.202*	0.153*	0.805**	0.857**	0.895**	0.731**	1

Note: **correlation significant at P < 0.01, *correlation significant at P < 0.05 level.

ST, surface temperature; MC, moisture content; TOC, total organic carbon; TN, total nitrogen; MBC, microbial biomass carbon and MBN, microbial biomass nitrogen.

**Table 2:** Multiple linear regression analysis of GHG fluxes on the physico-chemical indicators.

GHG	Unstandardized coefficient						Intercept	R^2^	*P*-value
	ST	MC	TOC	TN	MBC	MBN			
CO_2_	50.824 (<0.0001)	8.761 (0.116)	2.824 (0.001)	−63.716 (0.0001)	−0.007 (0.838)	0.051 (0.751)	−198.114	0.6532	<0.0001
ln(CH_4_ + 0.2)	0.086 (<0.0001)	−0.022 (0.291)	−0.006 (0.059)	0.398 (<0.0001)	−0.0001 (0.378)	0.0001 (0.869)	−1.805	0.6879	<0.0001
ln(N_2_O + 0.01)	−0.008 (0.611)	−0.043 (0.051)	−0.012 (0.001)	0.416 (<0.0001)	−0.0002 (0.071)	−0.001 (0.041)	−0.651	0.328	<0.0001

ST, surface temperature; MC, moisture content; TOC, total organic carbon; TN, total nitrogen; MBC, microbial biomass carbon and MBN, microbial biomass nitrogen. Note: values in the parenthesis are the p values of indicators in the model.

**Table 3:** Model estimates of average GHG fluxes for urine patches in each season.

Model^a^	ln(CH_4_ + 100) = 0.013 W^b^ + 3.51	ln(CO_2_) = 0.085T^c^ − 0.011 W + 6.31	ln(N_2_O) = 0.167 T + 0.079 W −1.06
	CH_4_ (μg m^−2 ^h^−1^)	CO_2_ (mg m^−2 ^h^−1^)	N_2_O (μg m^−2 ^h^−1^)
Early growing season	−38.5 ± 1.9	1292.1 ± 82.7	214.0 ± 32.0
Peak growing season	−39.6 ± 2.3	1364.6 ± 63.7	252.6 ± 54.3
Non-growing season	−35.1 ± 2.6	327.2 ± 13.4	25.6 ± 6.3

Note: ^a^Model from Lin *et al*.^14^; ^b^W is the water filled-pore space (WFPS) at 0–100 mm; ^c^T is the soil surface temperature at 0–50 mm.

**Table 4:** Total annual global warming potential (CO_2_-eq., tonnes) of a typical family farm grazing yaks on the Qinghai-Tibetan Plateau.

Season	GWP (CH_4_)	GWP (CO_2_)	GWP (N_2_O)	Total GWP
Early growing season	12.1 (−10.1%)	−145.6 (121.8%)	13.9 (−11.6%)	−119.6 (−35.8%)
Peak growing season	23.1 (−15.7%)	−224.8 (153.0%)	54.7 (−37.2%)	−147.0 (−44.0%)
Non-growing season	35.4 (5.9%)	484.3 (80.6%)	81.1 (13.5%)	600.7 (179.8%)
Total GWP	70.6 (21.1%)	113.83 (34.1%)	159.8 (44.8%)	334.2

Note: Data are the average for 9 farms with mean area 83 ha and stocking rate 1.05 yaks ha^−1^. Based on global warming potential 25 times CO_2_ for CH_4_ and 298 times CO_2_ for N_2_O^1^, the values inside parenthesis are the percentage by each season; Positive values mean emissions, and negative values mean uptake.
