# Supplementary material for: Key sources and seasonal dynamics of greenhouse gas fluxes from yak grazing systems on the Qinghai-Tibetan Plateau
Source: Sci Rep. 2017 Jan 20;7:40857. doi: 10.1038/srep40857 (PMC5247759; doi:10.1038/srep40857)
Supplement: Supplementary Information [file srep40857-s1.pdf]

Supplementary information for:

Key sources and seasonal dynamics of greenhouse gas fluxes from yak grazing systems on the Qinghai-Tibetan Plateau

Yang Liu[1], Caiyu Yan[1], Cory Matthew[2], Brennon Wood[2], Fujiang Hou[1]*

[1]State Key Laboratory of Grassland Agro-ecosystems, College of Pastoral Agriculture Science and Technology, Lanzhou University, Lanzhou, 730020, China.

[2]Institute of Agriculture and Environment, Massey University, Private Bag 11-222, Palmerston North, New Zealand.

*Correspondence and requests for materials should be addressed to F.H. (email: cyhoufj@lzu.edu.cn)

Supplementary Figure S1 – S2

Supplementary Table S1

Supplementary Fig. S1 Relational diagram of GHG sources on the Qinghai-Tibetan Plateau yak grazing systems. Major sources of GHG emissions from the soil-plant-animal system that were not directly measured in this study are depicted in grey.

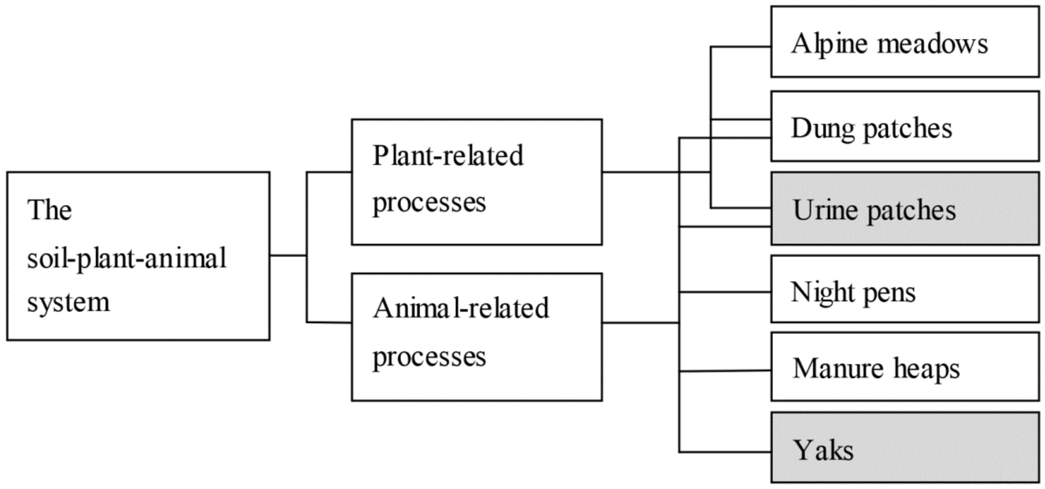

Supplementary Fig. S2 Daily mean temperature and precipitation on the Qinghai-Tibetan Plateau for 2013.

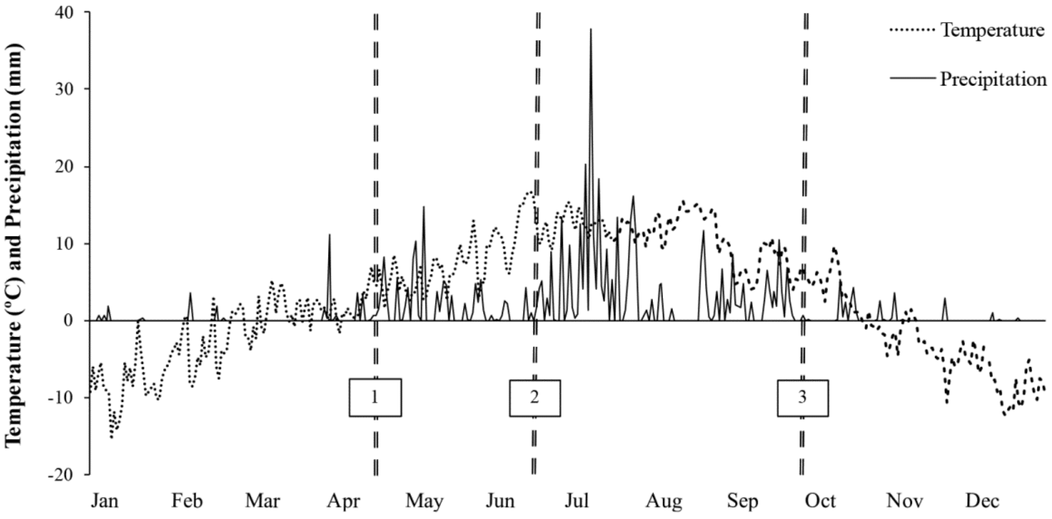

Table S1 Summary details for the nine yak grazing family farms.

Farm summary	Min	Max	Average (Stddev)
Household size	4	9	6 ± 2
Farm area (ha)	47	127	83 ± 29
Yak population (head)	44	130	87 ± 24
Dung patches area (ha)	0.9	2.2	1.5 ± 0.4
Urine patches area (ha)	1.0	2.7	1.7 ± 0.6
Night pens area (m^2)	1888	4704	2938 ± 837
Manure heaps footprint (m^2)	85	203	151 ± 48
